# Drowning

**Published:** 1872-08

**Authors:** 


### DROWNING

Is death by smothering. No air can get to the lungs, any more than when a pillow is pressed over the face, or a rope around the neck, closing the windpipe. The very first thing to be done is to send for a physician. The next, right on the shore, to take off all the clothing, wipe dry, wrap up the body in a blanket, and as quick as possible clear out the nose and mouth of any water or other obstruction there, and take means to get all the water you can out of the lungs, so that the air can take its place. While some are doing this, let others get bottles of hot water to put under the arm-pits and to the feet, so as to invite back warmth.

Turn the face downwards, with one arm supporting the forehead, so as to keep the nose and mouth from being buried in the sand, or dust, or mattress, or whatever else the person is lying upon. This also enables the tongue to fall forward, leaving the windpipe free, and the water falls naturally out of the mouth when any comes up from the lungs.

If breathing begins, assist the circulation of the blood by putting the hands under the blanket, and rubbing upwards, so as to keep the blood along towards the heart and lungs, where it will be warmed. Hot flannels and hot bricks should be applied, or hot sand in bags, to the soles of the feet, to the thighs, pit of stomach, as well as in the arm-pits. If now the patient can be got into the house, put him in the airiest, dryest, warmest room, windows open, giving every five minutes at first a tea-spoonful of spirits, or a table-spoon of wine, or hot tea or coffee. Keep in bed, and encourage sleep as much as possible.

But if there is little or no breathing, turn the patient on one side, — the right is better, — put snuff or hartshorn to the nose, or tickle it with a feather, or put the feather down the throat, for it excites cough; it will help to clear the lungs. Dash hot water, then cold water, on face and chest, and repeat from time to time with scarcely an appreciable interval for the first minute or two, but desist as soon as a convulsive attempt at breathing is noticed. If this does not seem to avail, put the patient on his face again, supporting the forehead with his arm as before; a folded coat or pillow or any bundle, even a log or a stone, under his belly; turn him gently on one side, then rapidly on his face; repeat this as rapidly as you can, at least fifteen times in a minute, keeping a firm pressure all the while between the shoulder-blades, behind and below them. The object of all this is to bring about something like the artificial mode of breathing, for when the patient is on his side the air will enter the lungs; when on his face, it naturally comes out. While all this is going on, apply well warmed flannels to feet and hands, and keep them dry, because dampness, by evaporation, carries heat away from the system rapidly, while the object should be to keep in it every atom of heat possible.

If this does not answer within five minutes, try another plan. Put the patient on his back, a small pillow or other bundle under his shoulders. Draw out the tongue, and run a string around it, so as to keep it out, thus leaving the windpipe uncovered. Take the arms above the elbows and draw them up steadily above the head. Keep them there two seconds. This expands the chest, and the air rushes into the lungs. Then draw the arms down to the sides, and close to them, pressing them against the sides of the chest for two seconds. This presses the air out of the chest. If this operation can be performed about fifteen times in a minute, the greatest chances are offered for a restoration of the breathing. The instant that is noticed, apply heat and warmth as instantly as possible, as above named.

If the person has been in the water for some time, or there are wounds or bruises; if the eyelids are half closed, and the pupils are larger than natural, the skin getting paler and colder, life may sometimes be restored after twenty minutes' apparent cessation of breathing. In one case, a patient came to life after eight hours were spent in the treatment. If there is the slightest lividity of the face, or a twitching of one of its muscles, or a convulsive movement in any limb or other part of the body, it is a sign of life still existing, and it may be invited back.

All intelligent persons, especially heads of families, owe it to themselves to read these instructions over and over again, until they are most thoroughly understood and remembered, with the reason for everything firmly fixed in the mind. It would not be out of the way to practice the motion on the living body, for any day and any hour there may be the sad occasion for using them on the body of some child or brother or sister or friend.

A small dog was put into a tub of water. The instant the head went under, a rapid inspiration of water was taken, and then a jerking expiration, which carried out a considerable amount of air. Thus the water took the place of the air in the lungs. No further effort to breathe was noticed, and in less than five minutes

THE DOG WAS DEAD.

The lips were closed, the top of the windpipe shut, and very little water was found in the branches of the windpipe.

A man fell into a well, and went under the water. He had his senses all about him, and he found himself trying to breathe out all the air he could, and aiming to prevent himself from swallowing water; for the next relief to getting fresh air into the lungs, is that of getting the old air, that which has been used, and is foul, out. Soon after his recovery, he vomited sand and mud. A young girl who was in the well with him, used no precautions, and convulsively sucked muddy sand into her lungs, which inflamed them, and for several days she was spitting up sandy mud from time to time.

These things show that death by drowning is simply a suffocation. The water in the windpipe is that taken in by the first convulsive effort at drawing in the breath; the lungs being supplied with no new air, cease to act; the pulse ceases to beat; the heart stops, and the man is dead. This mode of death is called

ASPHYXIA, which means literally "without pulse."

Sometimes it is a matter of very great consequence to identify a dead body, and to this end it is necessary, if the features are changed, to restore them as much as possible to what they were at the instant of death. This is done in a measure, sometimes, thus: Cover the cleansed body in water; pass through it a stream of chlorine gas. This whitens the skin, improved by adding some common salt to the water, and injecting tincture of steel into some of the blood-vessels of the face.

As lives are lost every summer at watering places, these suggestions are seasonable, and ought to be kept about the person, or within convenient reach.

If a person is struggling in the water, approach him, if possible, from behind, and raise the head only out of the water by the hair; and as the body loses a third or more of its weight when it is in the water, it requires but little strength to keep the head out of water, and to drag the body to the shore. For the same reason, it does not hurt the person much who is thus held up and dragged to the shore.

The grasp of a drowning person is so unreasoning and so convulsive, that every effort should be made by the rescuer to escape his clutches.

Persons in the water who cannot swim, can keep their heads above the surface for a considerable time by commencing at once a vigorous and quick treading operation, with both arms extended, and the palms of the hands resting on the water, for, small as they are, some support is afforded.

At other times, a person who has some presence of mind can support himself for half an hour in the water, and even longer, by throwing himself on his back, clasping both hands under him, and throwing his face upward upon the water, his feet extended, so that the nose only is out of the water. He can thus float for a considerable time without the expenditure of much strength.

If a person breaks through the ice, as in

SKATING, or in any other way, help is best given, and with greatest safety, by pushing a long rail or board or log of wood on the ice, until it reaches over the edge where it is broken, up to the person in the water, who can hold on to the end, and by this assistance can crawl out upon the edge of the ice, which might not be strong enough to bear both the rescued and the rescuer. If no bit of wood is at hand, then the rescuer might lie down on the ice, flat on his belly, and crawl towards the edge, keeping as large a part of the body on the ice as possible, for the strain on the ice is less in proportion to the surface to be supported. If more than one person is present to help save, let one go forward as before, and the next one hold on to his heel.

These things should be thought over carefully by all, that when there is occasion for them, the mind should feel at home in directing their execution.

